# Place Aux Dames

**Published:** 1905-03

**Authors:** 


### "PLACE AUX DAMES."

Our esteemed contemporary, the *Medical News*, has deserved well of posterity by directing attention to the observation that, in addition to the well-known risk of superficial burns, the vocation of the radiographer exposes him to the more insidious danger of damage to the spermatogenetic function. *Vetat pudor*, but a sense of justice to thousands yet unborn impels us to disclose the fact that, according to the revelations of a recent investigator, woman has still greater cause to fear this fell destroyer of germ cells than her tougher congener. Even the sheltered seclusion of the ovary affords no protection to its precious freight against the baleful emanations fathered by the German physicist. Halberstaedter, in the *Berliner klinische Wochenschrift* of January 16, describes experiments on rabbits which lead him to conclude that the function of the ovary is even more susceptible to the deleterious effect of the Roentgen ray than is that of its male analogue. So real does the danger seem to this author, that he recommends that prophylactic measures be carried out to guard against injury to the nurses in attendance in X-ray laboratories, by the use of suitable protective appliances, and that in treating abdominal conditions in women by the rays, the risk to the ovaries should always be kept in mind. Three sets of female rabbits were used in the experiments. The first series was rayed for half an hour on two occasions several days apart, while the second set was exposed twice as often. In each instance the animals were placed on the back during the treatment, with the right half of the abdomen protected by a plate of lead. At the autopsy about two weeks later, in every instance it was found that the left ovary was much smaller than the right, usually only half as large, and was devoid of Graafian follicles. A third series of tests was made in which the condition of the ovaries before treatment was established by laparotomy and inspection, so that accidental variation between the two sides could be excluded. Microscopical examination of the organs showed that ten days after the treatment the number of Graafian follicles was greatly reduced, while at the expiration of fifteen days they had all disappeared. In the animals of the first series primordial follicles and primitive ova were still distinguishable, so that a return to function was possible, but in the ovaries of the second series, destruction was so complete, that it seemed more than doubtful that new Graafian follicles could ever be produced. No doubt, when the results of Halberstaedter's experiments become known, the disciples of Malthus, whose advertisements appear so prominently in the newspapers, will find the installation of X-ray apparatus a profitable addition to the equipment of their "sanatoria."—*Medical Record.*

